# Occupational factors and miscarriages in the US fire service: a cross-sectional analysis of women firefighters

**DOI:** 10.1186/s12940-021-00800-4

**Published:** 2021-11-08

**Authors:** Alesia M. Jung, Sara A. Jahnke, Leslie K. Dennis, Melanie L. Bell, Jefferey L. Burgess, Nattinee Jitnarin, Christopher M. Kaipust, Leslie V. Farland

**Affiliations:** 1 Department of Epidemiology and Biostatistics, Mel and Enid Zuckerman College of Public Health, University of Arizona, 1295 N Martin Ave, Tucson, AZ 85724 USA; 2 Center for Fire, Rescue, & EMS Health Research, NDRI-USA, Leawood, KS USA; 3 Department of Community, Environment and Policy, Mel and Enid Zuckerman College of Public Health, University of Arizona, Tucson, AZ USA; 4 Department of Obstetrics and Gynecology, College of Medicine-Tucson, University of Arizona, Tucson, AZ USA

**Keywords:** Firefighters, Occupational health, Women’s health, Reproductive health, Epidemiology, Miscarriage, Spontaneous abortion

## Abstract

**Background:**

Evidence from previous studies suggests that women firefighters have greater risk of some adverse reproductive outcomes. The purpose of this study was to investigate whether women firefighters had greater risk of miscarriage compared to non-firefighters and whether there were occupational factors associated with risk of miscarriage among firefighters.

**Methods:**

We studied pregnancies in the United States fire service using data from the Health and Wellness of Women Firefighters Study (*n* = 3181). We compared the prevalence of miscarriage among firefighters to published rates among non-firefighters using age-standardized prevalence ratios. We used generalized estimating equations to estimate relative risks (RRs) and 95% confidence intervals (CIs) between occupational factors (employment (career/volunteer), wildland firefighter status (wildland or wildland-urban-interface/structural), shift schedule, fire/rescue calls at pregnancy start) and risk of miscarriage, adjusted for age at pregnancy, education, gravidity, BMI, and smoking. We evaluated if associations varied by age at pregnancy or employment.

**Results:**

Among 1074 firefighters and 1864 total pregnancies, 404 pregnancies resulted in miscarriages (22%). Among most recent pregnancies, 138 resulted in miscarriage (13%). Compared to a study of US nurses, firefighters had 2.33 times greater age-standardized prevalence of miscarriage (95% CI 1.96–2.75). Overall, we observed that volunteer firefighters had an increased risk of miscarriage which varied by wildland status (interaction *p*-value< 0.01). Among structural firefighters, volunteer firefighters had 1.42 times the risk of miscarriage (95% CI 1.11–1.80) compared to career firefighters. Among wildland/wildland-urban-interface firefighters, volunteer firefighters had 2.53 times the risk of miscarriage (95% CI 1.35–4.78) compared to career firefighters.

**Conclusions:**

Age-standardized miscarriage prevalence among firefighters may be greater than non-firefighters and there may be variation in risk of miscarriage by fire service role. Further research is needed to clarify these associations to inform policy and decision-making.

**Supplementary Information:**

The online version contains supplementary material available at 10.1186/s12940-021-00800-4.

## Background

In 2018 there were over 1.1 million estimated firefighters in the United States (US), 8% of whom were women [1]. Firefighters face specific occupational exposures (toxic substances and physical hazards) that may influence risk of adverse health outcomes, including cardiovascular diseases [2], respiratory diseases [3], certain cancers [4, 5], and infertility [6]. Firefighter research to date has been restricted primarily to male firefighters, and the health of women firefighters is critically understudied. Moreover, previously conducted studies have generally focused on career firefighters, who account for 33% (4% women) of the workforce, but may face different occupational exposures than volunteer firefighters (11% women) [1].

Health and safety issues among women in the fire service were first identified in the early 1990’s, but many of those concerns remain relevant and unanswered today. Previous research suggests that women firefighters are at greater risk of adverse reproductive health conditions [7, 8]. Career firefighters in the US have been suggested to have higher self-reported prevalence of pregnancy loss (miscarriages and stillbirths combined) compared to the general population [7]. The mechanisms associated with this potential increased risk in firefighters are hypothesized to be related to occupational exposures (e.g. wildfire smoke exposure, polycyclic aromatic hydrocarbons (PAHs), shift work, per-and polyfluoroalkyl substances (PFAS), and high environmental temperatures) that have been associated with adverse reproductive outcomes in other populations [9–13]. These occupational exposures are also assumed to vary between career firefighters and volunteers as well as between wildland or wildland-urban-interface (WUI, the transitional zone between unoccupied land and human development- an area where a built environment meets or intermingles with a natural/undeveloped environment) firefighters and structural firefighters.

While prior studies have consistently suggested that women firefighters are at increased risk of adverse reproductive outcomes, significant gaps in our understanding remain. It is not clear whether differences observed among career firefighters persist among different types of firefighters, such as career vs volunteer firefighters or wildland/WUI firefighters vs structural firefighters. Second, it is not known which occupational factors could potentially be associated with miscarriages. In our analysis, we analyzed data from a US cohort of women firefighters (*n* = 1074) to investigate the prevalence of miscarriage compared to non-firefighters and to assess potential occupational factors among firefighters that could have contributed to risk.

## Methods

### Study participants

The study sample was selected from a cohort of US women firefighters who participated in the Health and Wellness of Women Firefighters Study, developed and implemented by the Center for Fire, Rescue & EMS Health Research of NDRI-USA. The Health and Wellness of Women Firefighters Study was intended to examine the work environment, health, and perceived experiences of women firefighters in the fire service. Participants completed web-based surveys in 2017 and 2019. All study protocols and materials were approved of by the NDRI-USA Institutional Review Board and all participants provided informed consent. Details about the study recruitment and methodology have been reported previously [14]. Briefly, study recruitment was open to all women firefighters in the US and Canada above the age of 18. Due to the low prevalence of women within fire departments and the lack of a national, centralized firefighter database, the study utilized snowball sampling, a type of non-probability sampling where current participants recruit additional participants [15]. Initial participants were identified via affinity group email lists [7, 14, 16], including the International Association of Women in Fire and Emergency Service, the International Association of Fire Fighters, the National Volunteer Fire Council, and others. A $5 Amazon e-gift card was offered as an incentive to participate.

**Fig. 1:**
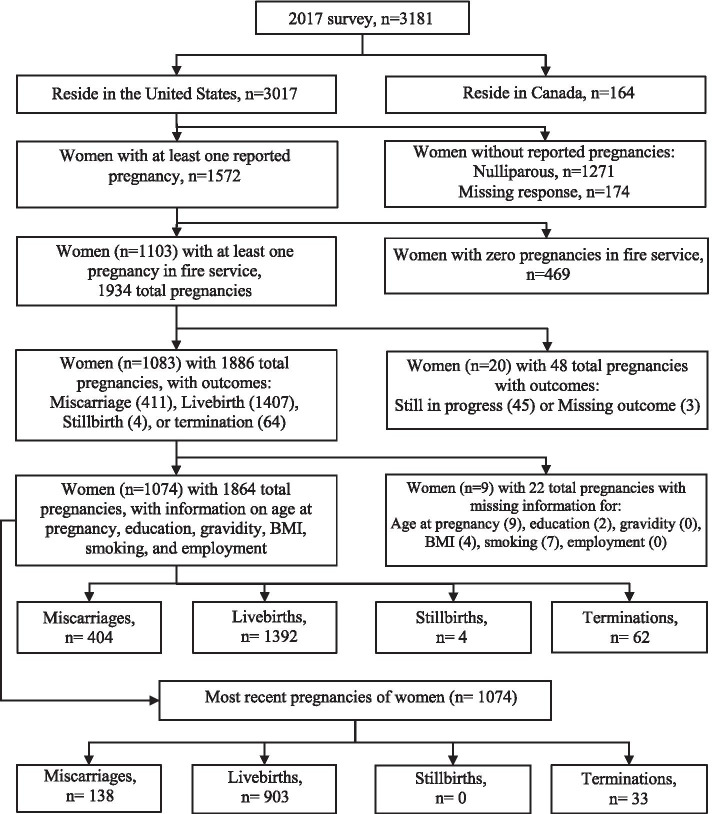
Women from the Health and Wellness of Women Firefighters Study in current analysis of miscarriages

For the current analysis, we examined firefighters who completed the 2017 survey (*n* = 3181), excluding Canadian firefighters (*n* = 163) given that strategies, tactics, and protective equipment may vary between the US and Canada (Fig. 1). Firefighters who had never been pregnant (*n* = 1271), were missing information on parity (*n* = 174), or who reported zero pregnancies while working in the fire service (*n* = 469) were excluded. Pregnancies that were still ongoing at the time of the 2017 survey (*n* = 45) or had a missing pregnancy outcome (*n* = 3) were excluded. Finally, women who had missing responses for age at pregnancy, education, gravidity, body mass index (BMI), and smoking were removed (*n* = 22). Based on these criteria, a total of 1074 women firefighters (34% of the original Health and Wellness of Women Firefighters Study population) were included in our analysis.

### Data collection

All data were self-reported and collected via the 2017 survey. The outcome of interest was miscarriage that occurred while working in the fire service. Firefighters were asked about pregnancies that had occurred over their lifetime (a maximum of ten pregnancies per woman, less than 1% reported ten pregnancies). For each pregnancy, participants reported if the pregnancy had occurred while working in the fire service and if the pregnancy had resulted in miscarriage, livebirth, stillbirth, or pregnancy termination. Pregnancies resulting in a miscarriage were compared to pregnancies assumed to be still at risk (livebirths, stillbirths, or pregnancy terminations).

The exposures of interest included firefighter types and work practices, based on status in 2017. Employment status (career or volunteer firefighter) was determined by asking “Are you primarily a career or volunteer firefighter?” Career firefighters can be generally considered full-time uniformed firefighters, regardless of responsibilities, which may include fire suppression, administration, or other tasks. Volunteer firefighters are generally considered part-time firefighters, who may serve as on-call or volunteer responders. Whether or not volunteer firefighters received any monetary compensation or health coverage through their work as volunteer firefighters was assumed to vary but was not measured in this study. Wildland firefighter status (wildland/WUI firefighter or structural firefighter) was determined by asking “Are you a wildland firefighter?”, with possible responses of “Yes, wildland firefighter only”, “Yes, I do wildland firefighting in addition to working for a career/volunteer fire department”, and “No”. While wildland firefighters respond exclusively to wildfires, WUI firefighters are generally those who may respond to wildfires, WUI fires, and structural fires during their service. Occupational exposures are thought to vary by employment and wildland firefighter status because of differences including but not limited to their total time spent at fires, exposures to different combustion products formed in smoke, and the equipment utilized and activities preformed [17, 18]. Career firefighters were also categorized as working for either > 24 consecutive hours at a time or working < 24 consecutive hours at a time. Working fire/rescue calls at pregnancy start (“Were you actively running fire or rescue calls when you found out you were pregnant for your X^th^ pregnancy?”, yes or no) was also assessed for each pregnancy.

Other variables of interest included age at pregnancy, gravidity (yes previously pregnant or not), race (white or other racial minority groups (Black, Asian, Native Hawaiian or other Pacific Islander, American Indian or Alaska Native, or other), hereafter referred to as other), Hispanic ethnicity (yes or no), highest level of education completed (some college or less, or college and above), BMI (< 30 kg/m^2^ or ≥ 30 kg/m^2^), smoking (current/former or never smoker), and presence of policies regarding pregnancy and/or maternity (yes or no). Education, BMI, smoking, and presence of pregnancy or maternity policies were assessed as current status in 2017. BMI (kg/m^2^) was calculated using self-reported height and weight in 2017. Smoking status (current, former, or never) was categorized using smoking history questions that assessed whether participants had smoked more than 100 cigarettes in their lifetime and whether they had smoked in the past 30 days [16]. Current smokers were those who reported that they had smoked more than 100 cigarettes in their lifetime and had smoked in the past 30 days. Former smokers were those who reported they had not smoked in the past 30 days but had smoked more than 100 cigarettes in their lifetime. Never smokers reported smoking less than 100 cigarettes in their lifetime. No detailed information regarding pregnancy or maternity policies was collected.

### Statistical analysis

Differences in characteristics of firefighters reported during the 2017 survey were assessed using two-sample t tests (continuous variables) and Chi-square tests (categorical variables). A *p*-value < 0.05 was considered statistically significant. Age-at-pregnancy standardized prevalence ratios (aSPRs), and 95% confidence intervals (95% CIs) were calculated using published indirect age-standardization methods and rates taken directly from two previously published analyses of US non-firefighters for comparison [19, 20]. We calculated aSPRs of miscarriage during most recent pregnancy (that resulted in miscarriage or livebirth) compared to a cohort of nurses enrolled in the Nurses’ Health Study II (NHSII) [19]. In 2001, NHSII assessed self-reported most recent pregnancies between 1993 and 2000 of women nurses and included pregnancies that resulted in either a self-reported miscarriage or a livebirth in their analysis. We also calculated aSPRs of miscarriage among most recent pregnancies that resulted in miscarriage, still birth, or livebirth compared to a cohort of California women in a prepaid health plan [20], as it was used as a comparison population in previously published research on firefighter health, where it was selected from among a limited number of US-based studies available at the time and examined age-stratified miscarriage risk among women assumed to be representative of the general population [7]. The California study assessed incidence of diagnosed miscarriages (pregnancy loss between gestational week 6 and 20) among clinically recognized pregnancies of women. Women who attended a prenatal visit and were less than 13 weeks pregnant were eligible for enrollment and those who consented to participate were followed throughout their pregnancies between February 1990 and September 1991, including pregnancies that resulted in miscarriage, stillbirth, or livebirth.

To compare occupational factors among our cohort of firefighters and risk of miscarriage, we used generalized estimating equations (GEE) with a Poisson distribution, log link function, exchangeable working correlation matrix, and sandwich variance estimators to directly estimate relative risks (RRs) and 95% CIs [21]. GEE was utilized to account for correlation between multiple pregnancies participants could contribute while working in the fire service. A binomial distribution was initially specified, however, due to observed non-convergence under the binomial distribution, a Poisson distribution was utilized in our final models, as has been previously suggested [21, 22]. A log link function was chosen to allow us to directly estimate RRs instead of odds ratios. Miscarriages may account for 10–20% of clinically recognized pregnancies [23] and odds ratios may overestimate RRs when the outcome is not rare (< 10%). So, to avoid the potential exaggeration and impact of our findings to the fire service we estimated RRs [24, 25]. An exchangeable (or compound symmetric) structure assumes that any two pregnancies for the same firefighter have the same correlation, regardless of timing. Sandwich variance estimation, an error variance procedure, was utilized because these standard errors can remain valid even if the correlation structure is misspecified, if the sample size is large enough.

We investigated associations between occupational factors (employment status, wildland firefighter status, assigned shift schedule of career firefighters, and working fire/rescue calls at pregnancy start) and the risk of miscarriage across all pregnancies in the fire service. In GEE models we adjusted for age at pregnancy, gravidity, education, BMI, and smoking because they were previously associated with increased risk for miscarriage [26–29] and they were a priori hypothesized to be associated with occupational factors. We adjusted for age at pregnancy (Model 1), because age at pregnancy has been reported to be a significant risk factor for risk of miscarriage and many other reproductive outcomes [29–32]. Age at pregnancy was modeled as age at pregnancy and age at pregnancy squared because the association between maternal age at pregnancy and miscarriage has been reported to follow a J-shaped curve, with a steep increase in risk of miscarriage after 35 years of age in several studies [30, 33]. In Model 2 we also adjusted for gravidity, education, BMI, and smoking as categorical variables.

Participants with missing responses for pregnancy outcome, age at pregnancy, education, gravidity, BMI, or smoking were excluded from the main analyses. We considered effect modification between age at pregnancy (< 35 years or ≥ 35 years) and occupational factors on the risk of miscarriage given that women older than 35 are two times as likely to have a miscarriage compared to women less than 35 years old [34]. In addition, other studies have reported that age at pregnancy potentially modified associations between potential firefighter exposures (shift work, particulate matter less than 2.5 μm in diameter (PM_2.5_), PFAS) and risk of adverse reproductive outcomes, including miscarriage [13, 35, 36]. We also considered potential effect modification between employment status and wildland firefighter status on the risk of miscarriage due to the substantial overlap of employment and wildland firefighter designations in the fire service and because exposures that may influence risk of miscarriage potentially vary between wildfires and structural fires [1, 37, 38]. We also evaluated potential effect modification between employment status and working fire/rescue calls at pregnancy start, since annual call volumes differ between career and volunteer departments [1]. Effect modification was investigated by creating an interaction term for the occupational exposure and the potential modifying factor and then performing tests of heterogeneity by assessing Wald *p*-values [39]. Interaction terms were considered in separate models. Stratified models were presented when effect modification was present.

We performed sensitivity analyses to test the robustness of our findings. First, we assessed risk of miscarriage in two different scenarios: (1) among the *most recent* pregnancy in the fire service and (2) the *first* pregnancy in the fire service using Poisson regression models with sandwich variance estimators [22]. Second, multiple imputation with chained equations (MICE) was used to assess the sensitivity of our estimates to missing responses for age at pregnancy, education, gravidity, BMI, and smoking [40]. Imputation models contained all variables from the analytical model, the outcome, variables determined to be associated with missingness, and auxiliary variables correlated with variables to be imputed (determined using Kendall’s Tau > 0.20). Twenty complete datasets were imputed and analyzed and estimates were pooled using Rubin’s Rules [41]. Third, we excluded pregnancies resulting in stillbirth or termination from our analyses (only analyzing miscarriages and livebirths). Fourth, we removed firefighters who reported that they were wildland only firefighters and repeated GEE models to determine if inherent differences between wildland firefighting and other firefighting [37, 42] were large enough to preclude the combination of wildland and WUI firefighters into a singular analytical category. Fifth, we adjusted for previous miscarriage (yes or no) in regression models, due to the strong pattern of recurrence of miscarriages [33]. Finally, we included age at time of survey in 2017 in our adjusted GEE models, to evaluate if any inherent differences in age between sub-groups of the firefighters analyzed affected our estimates. Statistical analyses were performed using SAS v 9.4 software (SAS Institute, Inc., Cary, NC, USA).

## Results

Among 1074 women and 1864 pregnancies in our analysis, there were 404 (22%) miscarriages, 1392 (75%) livebirths, 4 (< 1%) stillbirths, and 62 (3%) pregnancy terminations (Fig. 1). When limited to most recent pregnancies, there were 138 (13%) miscarriages, 903 (84%) livebirths, and 33 (3%) pregnancy terminations. The women in our analysis were on average 38 years old at time of survey in 2017 (interquartile range (IQR) 34–45), mostly white (93%), and non-Hispanic (95%). Additionally, 19% had BMI ≥ 30 kg/m^2^, 5% were current smokers, and 19% were former smokers (Table 1). Most participants were married or in a partnership (77%) and more than half had an average household income greater than $75,000. The average number of pregnancies over the lifetime was 2 (IQR 1–3), 31% of our participants reported ever having experienced a miscarriage, and 16% reported history of previous preterm birth. The average of years since their last pregnancy in the fire service was 6 (IQR 2–13). Participants were mostly career firefighters (85%); 66% were structural firefighters, 31% were WUI firefighters, and 2% were wildland firefighters. More than half of participants held ranks of firefighter or cross-trained firefighter/paramedic (62%) and 80% of career firefighters reported that they had an assigned shift schedule that involved working 24 or more hours while on shift. More than 30% of women reported that their departments did not have policies regarding employee pregnancies or maternity leave. Though career and volunteer firefighters were similar, career firefighters had lower average BMI (*p* < 0.0001) and were less likely to be smokers (*p* < 0.0001). Similarly, wildland/WUI firefighters were younger at time of survey completion (*p* < 0.0001), had a lower BMI (*p* < 0.0001), and were less likely to be smokers (*p* < 0.0001) compared to structural firefighters (Table 1).

**Table 1:** Characteristics of firefighters at 2017 survey with at least one pregnancy, by employment and wildland status^a^

	Employment status		Wildland firefighter status		Total
	Volunteer *n* = 157	Career *n* = 917	Structural *n* = 709	Wildland/WUI *n* = 363	*n* = 1074
**Demographics in 2017**					
	**Median (IQR)**				
**Age at survey (years)**	37 (31–46)	38 (34–45)	41 (35–47)	34 (34–39)	38 (34–45)
**Total pregnancies**^**b**^	2 (1–3)	2 (1–3)	2 (1–3)	1 (1–2)	2 (1–3)
**Total livebirths**	1 (1–2)	1 (1–2)	2 (1–2)	1 (1–2)	1 (1–2)
	**N (%)**				
**Race, white**	148 (95)	845 (93)	643 (92)	349 (96)	993 (93)
**Hispanic ethnicity**	< 5	51 (6)	44 (6)	10 (3)	55 (5)
**BMI**					
≤ 24.9 kg/m^2^	48 (31)	479 (52)	254 (36)	272 (75)	527 (49)
25–29.9 kg/m^2^	51 (32)	295 (32)	287 (40)	58 (16)	346 (32)
≥ 30 kg/m^2^	58 (37)	143 (16)	168 (24)	33 (9)	201 (19)
**Highest completed education**					
Some college or less	102 (65)	538 (59)	370 (52)	269 (74)	640 (60)
College and above	55 (35)	379 (41)	339 (48)	94 (26)	434 (40)
**Marital status**					
Married or in partnership	107 (68)	721 (79)	515 (73)	312 (86)	828 (77)
**Household income**					
> $75,000	77 (49)	601 (66)	570 (81)	107 (29)	678 (63)
≤ $75,00	79 (51)	315 (34)	137 (19)	256 (71)	394 (37)
**Smoking status**					
Current	22 (14)	30 (3)	41 (6)	11 (3)	55 (5)
Former	47 (30)	155 (17)	165 (23)	37 (10)	202 (19)
Never	88 (56)	732 (80)	503 (71)	319 (87)	820 (76)
**History of miscarriage**	70 (45)	261 (28)	279 (39)	51 (14)	331 (31)
**History of pre-term birth**	135 (15)	36 (23)	136 (19)	34 (9)	171 (16)
**Menopause status**					
Premenopausal	103 (71)	693 (78)	494 (72)	302 (86)	796 (77)
Perimenopausal	17 (12)	91 (10)	85 (12)	22 (6)	108 (10)
Postmenopausal	25 (17)	109 (12)	105 (15)	28 (8)	134 (13)
**Occupational factors in 2017**					
	**Median (IQR)**				
**Years since most recent pregnancy in fire service**	8 (3–14)	5 (2–12)	8 (3–14)	2 (2–8)	6 (2–13)
	**N (%)**				
**Employment status**					
Volunteer	–	–	118 (17)	39 (11)	157 (15)
Career	–	–	591 (83)	324 (89)	917 (85)
**Wildland fire activity**					
No (structural)	118 (75)	591 (65)	709 (100)	–	709 (66)
Yes (wildland only)	< 5	22 (2)	–	26 (7)	26 (2)
Yes (WUI)	35 (22)	302 (33)	–	337 (93)	337 (31)
**Current rank**					
Firefighter	75 (48)	328 (36)	160 (23)	242 (67)	403 (38)
Firefighter/paramedic	30 (19)	225 (25)	212 (30)	43 (12)	255 (24)
Driver operator	7 (5)	77 (8)	66 (9)	18 (5)	84 (8)
Lieutenant	10 (6)	89 (10)	82 (12)	16 (4)	99 (9)
Captain	8 (5)	95 (10)	76 (11)	27 (7)	103 (10)
Chief	14 (9)	74 (8)	77 (11)	11 (3)	88 (8)
Paramedic	5 (3)	5 (1)	10 (1)	0 (0)	10 (1)
Other	8 (5)	23 (3)	26 (4)	5 (1)	31 (3)
**Shift schedule (career only)**					
24 h or more on shift	–	735 (80)	444 (76)	290 (90)	735 (80)
Less than 24 h on shift	–	179 (20)	144 (24)	34 (10)	179 (20)
**Department policy regarding pregnancy and/or maternity**					
Yes	57 (36)	712 (78)	461 (65)	306 (84)	769 (72)
No	72 (46)	173 (19)	204 (29)	41 (11)	245 (23)
Don’t know	28 (18)	32 (3)	44 (6)	16 (4)	60 (6)

*IQR* interquartile range, *BMI* Body mass index (kg/m^2^), *WUI* wildland urban interface

Responses may not sum to totals due to missing responses not shown here. Percentages may not sum to 100 due to rounding

^a^ All data were collected at time of survey in 2017. Unless otherwise noted, variable is self-reported in 2017

^b^ Includes pregnancies that resulted in either livebirth, miscarriage, stillbirth, or pregnancy termination

A total of 1041 women firefighters reported that their most recent pregnancy in the fire service resulted in a miscarriage or a livebirth. Compared to a cohort of US nurses [19], the age-at-pregnancy standardized prevalence of miscarriage, hereafter referred to as age-standardized prevalence, among firefighters in our study was 2.33 times greater (95% CI 1.96, 2.75) (Table 2). When stratified by employment status, the age-standardized prevalence of miscarriage was higher among volunteer firefighters (aSPR 4.90; 95% CI 3.47–6.72) than career firefighters (aSPR 1.94; 95% CI 1.58–2.37) when compared to non-firefighters. Stratified by wildland firefighter status, the age-standardized prevalence of miscarriage was greater for structural firefighters (aSPR 2.76; 95% CI 2.28–3.32) compared to non-firefighters. Compared to miscarriage rates among California women insured by Kaiser Permanente Medical Care Program [20], the age-standardized prevalence of miscarriage among women firefighters was not increased (aSPR 1.09; 95% CI 0.91–1.28) (Table 2). When stratified by firefighter designations, the age-standardized prevalence of miscarriage was increased among volunteer firefighters (aSPR 2.09; 95% CI 1.48–2.87) and structural firefighters (aSPR 1.38; 95% CI 1.14–1.65), but lower in wildland/WUI firefighters (aSPR 0.53; 95% CI 0.34, 0.80) and non-statistically significant among career firefighters (aSPR 0.92; 95% CI 0.75–1.28), compared to California women.

**Table 2:** Age-at-pregnancy standardized prevalence ratios comparing miscarriage among most recent pregnancy of firefighters to US non-firefighters

	Observed events	Expected events	aSPR (95% CI)
**Firefighters compared to women from a US cohort of nurses**^a^			
All firefighters (*N* = 1041)	138	59.2	2.33 (1.96–2.75)
Employment status			
Career (*n* = 892)	100	51.4	1.94 (1.58–2.37)
Volunteer (*n* = 149)	38	7.8	4.90 (3.47–6.72)
Wildland firefighter status			
Structural (*n* = 685)	115	41.6	2.76 (2.28–3.32)
Wildland/WUI (*n* = 354)	23	17.4	1.32 (0.84–1.98)
**Firefighters compared to California women belonging to a prepaid health plan**^b^			
All firefighters (*N* = 1041)	138	127.0	1.09 (0.91–1.28)
Employment status			
Career (*n* = 892)	100	108.8	0.92 (0.75–1.12)
Volunteer (*n* = 149)	38	18.2	2.09 (1.48–2.87)
Wildland firefighter status			
Structural (*n* = 685)	115	83.6	1.38 (1.14–1.65)
Wildland/WUI (*n* = 354)	23	43.2	0.53 (0.34–0.80)

*US* United States, *aSPR* age-at-pregnancy-standardized prevalence ratio, *CI* confidence interval, *WUI* wildland urban interface

^a^ Lawson et al., 2014 (DOI: 10.1016/j.ajog.2011.12.030). Study included 7482 women from the Nurses’ Health Study II who self-reported details about their most recent pregnancy (resulting in miscarriage or livebirth) while working as a nurse between 1993 and 2000

^b^ Slama et al., 2005 (DOI: 10.1093/aje/kwi097). Study included 5121 pregnancies (resulting miscarriage, stillbirth, or livebirth) belonging to California, US members of the Kaiser Permanente Medical Care Program, with a prenatal appointment between February 1990 and September 1991. Data were abstracted from medical records

In analyses of pregnancies in our cohort, we observed that employment status and wildland firefighter status were associated with risk of miscarriage (Table 3). Volunteer firefighters had a greater risk of miscarriage compared to career firefighters, but the magnitude of risk varied by wildland firefighter status (interaction *p*-value< 0.01). Among structural firefighters (*n* = 1376 pregnancies), volunteer firefighters had 1.42 times the risk of miscarriage (95% CI 1.11–1.80) compared to career firefighters. Among wildland/WUI firefighters (*n* = 485 pregnancies), volunteer firefighters had 2.53 times the risk of miscarriage compared to career firefighters (RR 2.53, 95% CI 1.35–4.78).

**Table 3:** Associations between occupational factors and risk of miscarriage among 1074 firefighters and 1864 pregnancies^a,b^

	Miscarriages N (%)		RR (95% CI) Model 1^c^	RR (95% CI) Model 2^d^
**Firefighter subgroups**				
Employment, stratified by wildland firefighter status				
Structural	No	Yes		
Career	891 (76%)	281 (24%)	1.00 (Ref.)	1.00 (Ref.)
Volunteer	140 (69%)	64 (31%)	1.41 (1.11–1.79)	1.42 (1.11–1.80)
Wildland/WUI	No	Yes		
Career	388 (90%)	41 (10%)	1.00 (Ref.)	1.00 (Ref.)
Volunteer	38 (68%)	18 (32%)	3.09 (1.76–5.42)	2.53 (1.35–4.78)
Interaction *p*-value ^e^			< 0.01	< 0.01
**Work practices**				
Shift schedule of career firefighters, stratified by age at pregnancy				
< 35 years old	No	Yes		
Less than 24 h on shift	232 (77%)	69 (23%)	1.00 (Ref.)	1.00 (Ref.)
24 or more hr. on shift	772 (84%)	144 (16%)	0.67 (0.50–0.90)	0.74 (0.55–0.99)
35+ years old	No	Yes		
Less than 24 h on shift	64 (78%)	18 (22%)	1.00 (Ref.)	1.00 (Ref.)
24 or more hr. on shift	211 (70%)	89 (30%)	1.29 (0.84–2.00)	1.28 (0.82–1.99)
Interaction *p*-value ^f^			0.01	0.02
Worked fire/rescue calls at pregnancy start	No	Yes		
No	138 (70%)	58 (30%)	1.00 (Ref.)	1.00 (Ref.)
Yes	1250 (79%)	340 (21%)	0.81 (0.60–1.09)	0.82 (0.61–1.11)

*RR* Relative risk, *CI* confidence interval, *BMI* Body mass index (kg/m^2^), *WUI* wildland urban interface

^a^ Generalized estimating equations models with Poisson distribution and sandwich variance estimators were used to estimate risk ratios and 95% CIs

^b^ 86 pregnancies had missing information for wildland status (3), shift schedule (5) and fire/rescue calls (78) and were not included in those models

^c^ Model 1 is adjusted for age at pregnancy, modeled as age at pregnancy and (age at pregnancy)^2^

^d^ Model 2 is additionally adjusted for highest education completed (some college/at least college degree), gravidity (yes/no), BMI (< 30 kg/m^2^ / ≥30 kg/m^2^), and smoking status (current or former/never). Highest education completed, BMI, and smoking status were measured in 2017 at time of survey. Gravidity was assessed for each pregnancy

^e^ *p*-value for interaction between employment and wildland firefighter status

^f^ *p*-value for interaction between shift schedule and age at pregnancy, among career firefighters

The association between shift schedule for career firefighters and risk of miscarriage varied by age at pregnancy (interaction *p*-value = 0.02). Among career firefighters who were < 35 years old at pregnancy (*n* = 1217 pregnancies), having a shift schedule of > 24 h was associated with a reduced risk of miscarriage (RR: 0.74, 95% CI 0.55–0.99) compared to shift schedules of < 24 h (Table 3). This association did not reach the threshold of statistical significance among career firefighters who were > 35 at the time of pregnancy (*n* = 382 pregnancies) (RR 1.28, 95% CI 0.82–1.99). Report of actively working fire/rescue calls at pregnancy start was not associated with risk of miscarriage. Results from our sensitivity analyses were consistent and supported our main analyses (Additional file 1, Supplemental Tables 1–6).

## Discussion

The findings from our study provide further evidence suggesting that there may be a greater risk of adverse reproductive health outcomes among some groups of women firefighters and preliminary evidence that firefighters may have an increased risk of experiencing a pregnancy that results in miscarriage. We observed that the age-standardized prevalence of miscarriage was greater among firefighters compared to an occupational cohort of US nurses and that the risk of miscarriage varied among roles in the fire service. When investigating occupational factors among firefighters, we observed that volunteer firefighters had a greater risk of self-reported miscarriage compared to career firefighters and that the strength of this association varied by wildland firefighter status.

We observed higher age-standardized prevalence of self-reported miscarriage among firefighters compared to a non-firefighting cohort of US nurses (NHSII) [19]. This comparison is interesting given that nurses are another occupational group of front-line public service workers whose jobs share some characteristics to firefighters such as stress and working long shift schedules. However, this association attenuated when compared to a cohort of insured California women [20]. Miscarriage can be challenging to measure, so this discrepancy may be influenced by differences in data sources and study designs. Specifically, our study utilized self-reported data from a cross-sectional study, the NHSII analysis utilized self-reported data from a prospective study, and the study of insured California women utilized abstracted medical record data from a prospective study [19, 20]. Though we would expect medically assessed miscarriages to be more conservative than self-report, the California study might have oversampled women with increased risk of miscarriages since participants were enrolled from those scheduling their first prenatal appointment between gestational weeks 6 and 13, and this potential oversampling may have attenuated an association towards the null. As suggested previously, women who initiated early prenatal care might have sought early care because of a threatened miscarriage or because they were at increased risk for miscarriage [43]. Our finding builds on previous studies that have also reported increased risk of adverse reproductive outcomes among firefighters compared to non-firefighters [7, 8]. Potential mechanisms by which firefighters may have greater risk of miscarriages may involve potential occupational exposures (e.g., byproducts of combustion and certain PFAS), which have been studied in in other populations [11, 13]. Polycyclic aromatic hydrocarbons (PAHs), a byproduct of combustion have been found to influence miscarriage risk [11]. Firefighters may also be occupationally exposed to PFAS from PFAS-containing firefighting foams [44]. Maternal exposures to certain PFAS (perfluorononanoic acid (PFNA), perfluorodecanoic acid (PFDA), perfluorohexane sulfonate (PFHxS), and PFAS mixtures) have been associated with increased risk of miscarriage [13, 45]. Other occupational exposures, such as PM_2.5_ from wildfire smoke and high environmental temperatures, have been associated with other reproductive outcomes in non-firefighting populations, but there is limited research in relation to miscarriage risk [9, 12].

In our analysis of fire service pregnancies, we observed a greater risk of miscarriage in volunteer firefighters compared to career firefighters. Most firefighters in the US are volunteers, but firefighters employed in all-career or mostly-career fire departments typically respond to higher annual call volumes [1], therefore, volunteer firefighters are assumed to have fewer occupational exposures and lower risk of occupational diseases. However, compared to career firefighters, volunteers generally serve departments belonging to smaller communities. Because local taxes represent a large source of department revenue, volunteer departments may have less funding that can be allocated to the purchase of new technologies and upgraded equipment meant to prevent or minimize firefighter occupational exposures. Volunteer fire departments may also have reduced access to other resources (e.g., trained firefighters, support services/staff, protective equipment, training, occupational health services) compared to career departments. Partly due to this, volunteer fire departments may not allow pregnant firefighters to be assigned to administrative or light duty roles during pregnancy (a more commonly used practice in larger career departments that involves working shorter work shifts or positions where they will not be directly exposed to fires or fire suppression). This could have contributed a different set of occupational exposures among volunteer firefighters. In addition, women who join the volunteer fire service may hold full time jobs that may separately influence their risk of miscarriage. Given our findings related to miscarriage, and the fact that this is the first article on the reproductive health of volunteer women, more research on this population to understand what puts them at such increased risk is warranted.

Our results also suggest that the risk of miscarriage in the fire service may vary depending on wildland firefighter status. This association persisted even after adjusting for key risk factors of miscarriage for which wildland or WUI firefighters differed (younger age, lower BMI, fewer smokers) compared to structural firefighters. However, wildland or WUI firefighters on average had fewer years in the fire service compared to structural firefighters. Because we lacked pregnancy-specific data and were unable to account for years of service, this may have affected our results. Firefighters may respond to 1) wildland fires exclusively (wildland firefighters), 2) wildland fires and fires of human structures and infrastructure (WUI firefighters), or 3) fires of human structures and infrastructure exclusively (structural firefighters). Wildland and WUI firefighters may be exposed to higher core temperatures, increased physical exertion, and increased exposure to smoke, as they regularly engage in activities not shared by structural firefighters (e.g. hiking, constructing fire-lines, use of certain equipment, prescribed burning), as well as spend greater time at fires heavily concentrated over the span of a fire season [17, 18, 46]. One study, that merged data from two nationwide investigations of mental health, observed that wildland firefighters reported greater risk of suicide compared to structural firefighters, which may indicate different or greater mental health burdens among this group [47]. This is relevant considering that prenatal psychological distress has been associated with an increased risk of miscarriage [48]. These factors may have led to increased cumulative occupational exposures, mental strain, and physical exertion contributing to thermal burden and physiological strain, which could have contributed to a greater risk for occupational injuries and diseases.

We also observed a potential association between shift schedule and miscarriage risk, which differed by age at pregnancy. Among participants who were younger at time of pregnancy (< 35 years), working 24 h or more hours per shift was associated with lower risk of miscarriage compared to working shorter shift schedules. A previous study of Norwegian nurses found that young age was associated with higher shift work tolerance, essentially the ability to adapt to shift work without adverse consequences such as sleepiness, fatigue, or impaired social functioning, and physical, and mental health [49]. It is possible that younger firefighters may have been more likely to have higher shift work tolerance or overall increased health and therefore may have been able continue working 24 h or more shifts longer during their pregnancy. Among participants who were older at time of pregnancy, this association was no longer statistically significant. Older age at pregnancy is an established risk factor for miscarriage [33], so among older women, shift work may not meaningfully influence risk given other strong risk factors. Among younger women, it is possible that if they had experienced adverse pregnancy outcomes or pregnancy complications during a previous pregnancy, they may have been more likely to choose administrative or light duty roles (not measured in this study), which may have biased our results.

To our knowledge, using data from the largest study of the health and wellness of women firefighters in the US to date, this is the first analysis of occupational factors and miscarriages among firefighters. Our study is also a novel investigation of reproductive health outcomes among volunteers and wildland or WUI firefighters, subgroups in the fire service that are often underrepresented in firefighter research. Despite the strengths of our study, there are important limitations to consider. Our age-standardized prevalence ratios should be interpreted with caution due to several limitations. First, while our method of indirect age-standardization accounted for differing age structures of our study sample and the two reference groups, it failed to account for other potential population level confounding factors. However, this is a limitation common to all age-standardization, including a previous study of cancer incidence and mortality among firefighters [4]. In our study, we compared report of miscarriage to rates taken from a study of US nurses enrolled in the NHSII [19], selected due to its analysis of another occupational cohort of women, and a study of insured California women [20], because it was used as a reference group in a previous study of firefighters by Jahnke et al., who selected the California study from the limited number of US-based studies of age-stratified miscarriage risk available at the time because the participants were assumed to be representative of the general population [7]. As discussed above, our comparisons to the study of California women may be limited by significant differences in the data sources, study design, and the women enrolled. Our comparison to the US nurses may be limited to a lesser extent as both are occupational cohorts of mostly non-Hispanic white women who reported working during their previous pregnancy and both studies relied on self-reported measures. Still, our findings do suggest that a more structured comparison of firefighters to non-firefighters is needed.

There could be potential selection bias by the firefighters who participated in our study. Snowball sampling is reliant on initial contacts identifying participants. Contacted firefighters may have been more social, well connected, more likely to be a member of a large fire service organization, or more receptive to research, which could have been affected by health history. It is also possible that occupational factors in this study were influenced by sociability, since participants may have been more likely to recommend peers within their departments or occupational networks. Additionally, women who experience miscarriages may be more likely to experience post-partum depression, anxiety, and stress, which can impact their sociability [50] and their desire to participate in health focused research. This potential bias may have a stronger effect on the association observed between employment (volunteer vs career) and risk of miscarriage in our study. Indeed, in our analysis, 15% of participants in our study were volunteer firefighters, while approximately 84% of women firefighters in the US are volunteers [1]. If the volunteers who participated in our study were more likely to have an adverse reproductive health history, this could have led to an overestimation of the association between volunteer firefighters and risk of miscarriage. However, this study did not emphasize reproductive health topics during enrollment promotion, so we expect differential participation to be minimal. Overall, our results may not be generalizable to the entire fire service and future research should consider how to increase study participation from firefighters belonging to more isolated departments and subgroups, particularly volunteer firefighters.

There is also the potential for misclassification from several sources. Because we lacked data on employment, wildland firefighter status, and shift schedule at the time of each pregnancy, we had to assume that current employment, wildland firefighter status, and shift schedule reported in 2017 was a valid proxy for each pregnancy analyzed. However, this misclassification would most likely have been non-differential and therefore, attenuate our effect estimates. It is also possible that firefighters may have transitioned between volunteer and career positions; potential reasons include improved job opportunities, relocation, or transition of a volunteer department to career or combination staffing. In addition, the current study did not collect detailed information about potential work restrictions (e.g. light duty roles) taken during these pregnancies which could have exaggerated the association we observed between employment status and risk of miscarriage, because volunteer firefighters may be less likely to be able to work light duty roles than career firefighters. However, this effect was most likely not large since most miscarriages occur within the first trimester of pregnancy within the first 12 weeks of pregnancy and though published guidance on when to switch firefighters to light-duty is limited, one recommendation is to do so by gestational week 20 [51], near the cutoff of a defined miscarriage. The study also did not collect information on specific occupations volunteers might have engaged in primarily which could have independently influenced their risk for miscarriage. For wildland firefighter status, identifying as a wildland or WUI firefighter during an index pregnancy may not equate to actively participating in either fire suppression or a fire season. These firefighters might have reported pregnancies that occurred outside of wildfire season. Or firefighters who were trying to conceive or were aware of their pregnancy might have chosen to not participate in the fire season. This would have attenuated observed associations. There is a paucity of research on the effects of cumulative wildfire exposure on reproductive health outcomes, so it is possible that pregnancies that occurred outside of wildfire season could still be impacted by previous work. Future research should incorporate information on cumulative fireground exposures.

Misclassification of miscarriage may also be a limitation of our study, as recall of miscarriage is challenging. Between 10 and 20% of clinically recognized pregnancies are estimated to result in miscarriage though the true incidence may be closer to 30% [23, 52]. This outcome misclassification would most likely be non-differential with respect to the exposures and therefore, most likely would have attenuated our effect estimates. Additionally, participants were asked to report pregnancy details for multiple (up to ten) previous pregnancies which may lead to difficulties with recall. However, we would expect any misclassification to be non-differential with respect to our exposures. Indeed, results from our sensitivity analyses restricting to most recent pregnancy showed similar overall patterns compared to our main results (Table 3 and Supplemental Table 1).

Finally, due to the cross-sectional design of the study, the results of our analysis cannot be used to support a causal relationship between firefighter occupational factors and the risk of miscarriage, because of the complex temporality of the exposures and the outcome. However, our findings have identified several areas of focus for future research, including potentially higher risk among volunteers and wildland/WUI firefighters and the impact of shift schedules, that should be explored in future studies to substantiate our findings and clarify potential mechanisms.

## Conclusions

Consistent with previous research, our study provides further evidence that adverse reproductive outcomes may be elevated among firefighters compared to non-firefighters, and preliminary evidence that miscarriage risk may vary within the fire service. Our study also highlights future research opportunities to investigate the influence of additional firefighter occupational risk factors and mechanisms. Improving our understanding of miscarriages and other adverse reproductive outcomes in the fire service and the risk factors associated with them could be used to inform subsequent policy development, to inform personal decision-making, and to provide opportunities for public health interventions for women firefighters.

## Supplementary Information

**Additional file 1: Supplemental Tables 1–6.** Results of sensitivity analyses.

## Data Availability

The data generated or analyzed during the current study are not publicly available due to restrictions on sharing data, based on the consent forms and IRB application for this study, but are available from the authors upon reasonable request.

## Abbreviations

aSPR: Age-at-pregnancy standardized prevalence ratio
BMI: Body mass index
CI: Confidence interval
GEE: Generalized estimating equations
IQR: Interquartile range
MICE: Multiple imputation with chained equations
NHSII: Nurses’ Health Study II
PAH: Polycyclic aromatic hydrocarbons
PFAS: Per- and polyfluoroalkyl substances
PFDA: Perfluorodecanoic acid
PFHxS: Perfluorohexane sulfonate
PFNA: Perfluorononanoic acid
PM_2.5_: Particulate matter less than 2.5 μm in diameter
RR: Relative risk
WUI: Wildland-urban-interface
US: United States
