# Supplementary material for: Occupational factors and miscarriages in the US fire service: a cross-sectional analysis of women firefighters
Source: Environ Health. 2021 Nov 8;20:116. doi: 10.1186/s12940-021-00800-4 (PMC8573857; doi:10.1186/s12940-021-00800-4)
Supplement: Supplementary file 1 — Additional file 1: Supplemental Tables 1–6. Results of sensitivity analyses. [file 12940_2021_800_MOESM1_ESM.docx]

**Occupational factors and miscarriages in the US fire service: a cross-sectional analysis of women firefighters**

**Supplemental Tables**

Supplemental Table 1. Associations between occupational factors and risk of miscarriage during most recent and first pregnancies, among 1,074 firefighters 2

Supplemental Table 2. Associations between occupational factors and risk of miscarriage using multiple imputation with chained equations to impute missing variables, among 1,083 firefighters and 1,886 pregnancies 3

Supplemental Table 3. Associations between occupational factors and risk of miscarriage, restricted to miscarriages and livebirths, among 1,041 firefighters and 1,796 pregnancies 4

Supplemental Table 4. Associations between occupational factors and risk of miscarriage, excluding wildland firefighters, among 1,048 firefighters and 1,812 pregnancies 5

Supplemental Table 5. Associations between occupational factors and risk of miscarriage adjusted for previous miscarriages, among 1,053 firefighters and 1,837 pregnancies 6

Supplemental Table 6. Associations between occupational factors and risk of miscarriage adjusted for age at survey, among 1,074 firefighters and 1,864 pregnancies 7

| **Supplemental Table 1.** Associations between occupational factors and risk of miscarriage during most recent and first pregnancies, among 1,074 firefighters^a^ | | | | | | | | |
| --- | --- | --- | --- | --- | --- | --- | --- | --- |
|  | **Most recent pregnancy in fire service^b^** | | | | **First pregnancy in fire service^c^** | | | |
|  | Miscarriages  N | | RR (95% CI)  Model 1^d^ | RR (95% CI)  Model 2^e^ | Miscarriages  N | | RR (95% CI)  Model 1^d^ | RR (95% CI)  Model 2^e^ |
| **Firefighter subgroups** |  | | | |  | | | |
| Employment, stratified by wildland firefighter status |  |  |  |  |  |  |  |  |
| Structural | No | Yes |  |  | No | Yes |  |  |
| Career | 504 | 87 | 1.00 (Ref.) | 1.00 (Ref.) | 455 | 136 | 1.00 (Ref.) | 1.00 (Ref.) |
| Volunteer | 91 | 27 | 1.64 (1.10 - 2.42) | 1.54 (1.03 - 2.31) | 79 | 39 | 1.53 (1.23 - 208) | 1.54 (1.13 – 2.10) |
| Wildland/WUI |  |  |  |  |  |  |  |  |
| Career | 311 | 13 | 1.00 (Ref.) | 1.00 (Ref.) | 303 | 21 | 1.00 (Ref.) | 1.00 (Ref.) |
| Volunteer | 29 | 10 | 4.18 (1.53 - 11.44) | 3.29 (1.18 - 9.18) | 25 | 14 | 4.94 (2.51 - 9.70) | 3.40 (1.68 – 6.86) |
| **Work practices** |  | | | |  | | | |
| Shift schedule of career firefighters, stratified by age at pregnancy |  |  |  |  |  |  |  |  |
| <35 years | No | Yes |  |  | No | Yes |  |  |
| Less than 24 hr on shift | 106 | 15 | 1.00 (Ref.) | 1.00 (Ref.) | 120 | 34 | 1.00 (Ref.) | 1.00 (Ref.) |
| 24 or more hr on shift | 506 | 39 | 0.65 (0.37 - 1.14) | 0.71 (0.40 - 1.26) | 536 | 86 | 0.67 (0.46 - 0.97) | 0.73 (0.51 - 1.06) |
| 35+ years |  |  |  |  |  |  |  |  |
| Less than 24 hr on shift | 51 | 7 | 1.00 (Ref.) | 1.00 (Ref.) | 17 | 8 | 1.00 (Ref.) | 1.00 (Ref.) |
| 24 or more hr on shift | 152 | 38 | 1.55 (0.73 - 3.29) | 1.50 (0.70 - 3.21) | 85 | 28 | 0.78 (0.40 - 1.52) | 0.74 (0.37 - 1.46) |
| Worked fire/rescue calls at pregnancy start | No | Yes |  |  | No | Yes |  |  |
| No | 91 | 28 | 1.00 (Ref.) | 1.00 (Ref.) | 57 | 31 | 1.00 (Ref.) | 1.00 (Ref.) |
| Yes | 800 | 106 | 0.56 (0.38 - 0.82) | 0.61 (0.41 - 0.91) | 753 | 175 | 0.55 (0.40 - 0.76) | 0.60 (0.43 - 0.82) |
| RR= Relative risk; CI= confidence interval; BMI= Body mass index (kg/m^2^); WUI=wildland urban interface  ^a^ Generalized estimating equations models with Poisson distribution and sandwich variance estimators were used to estimate risk ratios and 95% CIs.  ^b^ 54 pregnancies had missing information for wildland status (2), shift schedule (3), and fire/rescue calls (49) and were not included in those models.  ^c^ 63 pregnancies had missing information for wildland status (2), shift schedule (3), and fire/rescue calls (58) and were not included in those models.  ^d^ Model 1 is adjusted for age at pregnancy, modeled as age at pregnancy and (age at pregnancy)^2^.  ^e^ Model 2 is additionally adjusted for education (some college/at least college degree), gravidity (yes/no), BMI (<30 kg/m^2^ / ≥30 kg/m^2^), and smoking status (current or former/never). Education, BMI, and smoking status were measured in 2017 at time of survey. Gravidity was assessed for each pregnancy. | | | | | | | | |

| **Supplemental Table 2.** Associations between occupational factors and risk of miscarriage using multiple imputation with chained equations to impute missing variables, among 1,083 firefighters and 1,886 pregnancies^a,b^ | | | | |
| --- | --- | --- | --- | --- |
|  | Miscarriages  N | | RR (95% CI)  Model 1^c^ | RR (95% CI)  Model 2^d^ |
| **Firefighter subgroups** |  | | | |
| Employment, stratified by wildland firefighter status |  |  |  |  |
| Structural | No | Yes |  |  |
| Career | 897 | 285 | 1.00 (Ref.) | 1.00 (Ref.) |
| Volunteer | 141 | 66 | 1.42 (1.12 - 1.81) | 1.43 (1.13 - 1.81) |
| Wildland/WUI |  |  |  |  |
| Career | 395 | 42 | 1.00 (Ref.) | 1.00 (Ref.) |
| Volunteer | 39 | 18 | 3.01 (1.72 - 5.29) | 2.48 (1.32 - 4.66) |
| **Work practices** |  | | | |
| Shift schedule of career firefighters, stratified by age at pregnancy |  |  |  |  |
| <35 years old | No | Yes |  |  |
| Less than 24 hr on shift | 236 | 70 | 1.00 (Ref.) | 1.00 (Ref.) |
| 24 or more hr on shift | 780 | 146 | 0.68 (0.50 - 0.91) | 0.75 (0.56 – 0.99) |
| 35+ years old |  |  |  |  |
| Less than 24 hr on shift | 65 | 19 | 1.00 (Ref.) | 1.00 (Ref.) |
| 24 or more hr on shift | 211 | 90 | 1.25 (0.82 - 1.90) | 1.24 (0.81 - 1.90) |
| Worked fire/rescue calls at pregnancy start | No | Yes |  |  |
| No | 139 | 60 | 1.00 (Ref.) | 1.00 (Ref.) |
| Yes | 1263 | 345 | 0.79 (0.59 - 1.06) | 0.78 (0.59 - 1.03) |
| RR= Relative risk; CI= confidence interval; BMI= Body mass index (kg/m^2^); WUI= wildland urban interface  ^a^ Generalized estimating equations models with Poisson distribution and sandwich variance estimators were used to estimate risk ratios and 95% CIs.  ^b^ 88 pregnancies had missing information for wildland status (3), shift schedule (5), and fire/rescue calls (79) and were not included in those models.  ^c^ Model 1 is adjusted for age at pregnancy, modeled as age at pregnancy and (age at pregnancy)^2^.  ^d^ Model 2 is additionally adjusted for education (some college/at least college degree), gravidity (yes/no), BMI (<30 kg/m^2^ / ≥30 kg/m^2^), and smoking status (current or former/never). Education, BMI, and smoking status were measured in 2017 at time of survey. Gravidity was assessed for each pregnancy. | | | | |

| **Supplemental Table 3.** Associations between occupational factors and risk of miscarriage, restricted to miscarriages and livebirths, among 1,041 firefighters and 1,796 pregnancies^a,b^ | | | | |
| --- | --- | --- | --- | --- |
|  | Miscarriages  N | | RR (95% CI)  Model 1^c^ | RR (95% CI)  Model 2^d^ |
| **Firefighter subgroups** |  | | | |
| Employment, stratified by wildland firefighter status^d^ |  |  |  |  |
| Structural | No | Yes |  |  |
| Career | 844 | 281 | 1.00 (Ref.) | 1.00 (Ref.) |
| Volunteer | 130 | 64 | 1.39 (1.09 - 1.77) | 1.39 (1.09 - 1.77) |
| Wildland/WUI |  |  |  |  |
| Career | 380 | 41 | 1.00 (Ref.) | 1.00 (Ref.) |
| Volunteer | 35 | 18 | 3.09 (1.80 - 5.32) | 2.53 (1.35 - 4.76) |
| **Work practices** |  | | | |
| Shift schedule of career firefighters, stratified by age at pregnancy |  |  |  |  |
| <35 years old | No | Yes |  |  |
| Less than 24 hr on shift | 216 | 69 | 1.00 (Ref.) | 1.00 (Ref.) |
| 24 or more hr on shift | 741 | 144 | 0.65 (0.49 - 0.87) | 0.73 (0.54 - 0.97) |
| 35+ years old |  |  |  |  |
| Less than 24 hr on shift | 62 | 18 | 1.00 (Ref.) | 1.00 (Ref.) |
| 24 or more hr on shift | 205 | 89 | 1.28 (0.83 - 1.97) | 1.27 (0.82 - 1.97) |
| Worked fire/rescue calls at pregnancy start | No | Yes |  |  |
| No | 138 | 58 | 1.00 (Ref.) | 1.00 (Ref.) |
| Yes | 1246 | 340 | 0.81 (0.60 - 1.09) | 0.83 (0.61 - 1.11) |
| RR= Relative risk; 95% CI= 95% confidence interval; BMI= Body mass index (kg/m^2^); WUI= wildland urban interface  ^a^ Generalized estimating equations models with Poisson distribution and sandwich variance estimators were used to estimate risk ratios and 95% CIs.  ^b^ 21 pregnancies had missing information for wildland status (3), shift schedule (5), and fire/rescue calls (14) and were not included in those models.  ^c^ Model 1 is adjusted for age at pregnancy, modeled as age at pregnancy and (age at pregnancy)^2^.  ^d^ Model 2 is additionally adjusted for education (some college/at least college degree), gravidity (yes/no), BMI (<30 kg/m^2^ / ≥30 kg/m^2^), and smoking status (current or former/never). Education, BMI, and smoking status were measured in 2017 at time of survey. Gravidity was assessed for each pregnancy. | | | | |

| **Supplemental Table 4.** Associations between occupational factors and risk of miscarriage, excluding wildland firefighters, among 1,048 firefighters and 1,812 pregnancies^a,b^ | | | | |
| --- | --- | --- | --- | --- |
|  | Miscarriages  N | | RR (95% CI)  Model 1^c^ | RR (95% CI)  Model 2^d^ |
| **Firefighter subgroups** |  | | | |
| Employment, stratified by wildland firefighter status |  |  |  |  |
| Structural | No | Yes |  |  |
| Career | 891 | 281 | 1.00 (Ref.) | 1.00 (Ref.) |
| Volunteer | 140 | 64 | 1.41 (1.11 - 1.79) | 1.42 (1.11- 1.81) |
| WUI |  |  |  |  |
| Career | 350 | 31 | 1.00 (Ref.) | 1.00 (Ref.) |
| Volunteer | 35 | 17 | 3.35 (1.77 - 6.33) | 3.24 (1.59 - 6.59) |
| **Work practices** |  | | | |
| Shift schedule of career firefighters, stratified by age at pregnancy |  |  |  |  |
| <35 years old | No | Yes |  |  |
| Less than 24 hr on shift | 213 | 60 | 1.00 (Ref.) | 1.00 (Ref.) |
| 24 or more hr on shift | 760 | 143 | 0.70 (0.52 - 0.95) | 0.76 (0.56 - 1.03) |
| 35+ years old |  |  |  |  |
| Less than 24 hr on shift | 59 | 18 | 1.00 (Ref.) | 1.00 (Ref.) |
| 24 or more hr on shift | 209 | 89 | 1.22 (0.80 - 1.88) | 1.23 (0.80 - 1.90) |
| Worked fire/rescue calls at pregnancy start | No | Yes |  |  |
| No | 130 | 54 | 1.00 (Ref.) | 1.00 (Ref.) |
| Yes | 1218 | 333 | 0.83 (0.61 - 1.12) | 0.84 (0.62 - 1.13) |
| RR= Relative risk; 95% CI= 95% confidence interval; BMI= Body mass index (kg/m^2^); WUI= wildland urban interface  ^a^ Generalized estimating equations models with Poisson distribution and sandwich variance estimators were used to estimate risk ratios and 95% CIs.  ^b^ 85 pregnancies had missing information for wildland status (3), shift schedule (5), and fire/rescue calls (77) and were not included in those models.  ^c^ Model 1 is adjusted for age at pregnancy, modeled as age at pregnancy and (age at pregnancy)^2^.  ^d^ Model 2 is additionally adjusted for highest education completed (some college/at least college degree), gravidity (yes/no), BMI (<30 kg/m^2^ / ≥30 kg/m^2^), and smoking status (current or former/never). Education, BMI, and smoking status were measured in 2017 at time of survey. Gravidity was assessed for each pregnancy. | | | | |

| **Supplemental Table 5.** Associations between occupational factors and risk of miscarriage adjusted for previous miscarriages, among 1,053 firefighters and 1,837 pregnancies^a,b^ | | | | |
| --- | --- | --- | --- | --- |
|  | Miscarriages  N | | RR (95% CI)  Model 1^c^ | RR (95% CI)  Model 2^d^ |
| **Firefighter subgroups** |  | | | |
| Employment, stratified by wildland firefighter status |  |  |  |  |
| Structural | No | Yes |  |  |
| Career | 881 | 279 | 1.00 (Ref.) | 1.00 (Ref.) |
| Volunteer | 135 | 60 | 1.38 (1.08 - 1.76) | 1.42 (1.11 - 1.81) |
| Wildland/WUI |  |  |  |  |
| Career | 384 | 41 | 1.00 (Ref.) | 1.00 (Ref.) |
| Volunteer | 35 | 18 | 3.20 (1.85 - 5.55) | 2.68 (1.41 - 5.09) |
| **Work practices** |  | | | |
| Shift schedule stratified by age at pregnancy |  |  |  |  |
| <35 years old | No | Yes |  |  |
| Less than 24 hr on shift | 228 | 67 | 1.00 (Ref.) | 1.00 (Ref.) |
| 24 or more hr on shift | 768 | 144 | 0.68 (0.50 - 0.91) | 0.74 (0.553 - 0.99) |
| 35+ years old |  |  |  |  |
| Less than 24 hr on shift | 63 | 18 | 1.00 (Ref.) | 1.00 (Ref.) |
| 24 or more hr on shift | 207 | 89 | 1.30 (0.84 - 2.00) | 1.22 (0.79 – 1.88) |
| Worked fire/rescue calls at pregnancy start | No | Yes |  |  |
| No | 132 | 56 | 1.00 (Ref.) | 1.00 (Ref.) |
| Yes | 1241 | 338 | 0.81 (0.60 - 1.10) | 0.82 (0.61 - 1.11) |
| RR= Relative risk; 95% CI= 95% confidence interval; BMI= Body mass index (kg/m^2^); WUI= wildland urban interface  ^a^ Generalized estimating equations models with Poisson distribution and sandwich variance estimators were used to estimate risk ratios and 95% CIs. 21 women and 26 pregnancies were removed from the original analysis sample due to missing information on history of previous miscarriage.  ^b^ 86 pregnancies had missing information for wildland status (3), shift schedule (5), and fire/rescue calls (78) and were not included in those models.  ^c^ Model 1 is adjusted for age at pregnancy, modeled as age at pregnancy and (age at pregnancy)^2^.  ^d^ Model 2 is additionally adjusted for previous miscarriage (yes/no/nulligravid), highest education completed (some college/at least college degree), gravidity (yes/no), BMI (<30 kg/m^2^ / ≥30 kg/m^2^), and smoking status (current or former/never). Education, BMI, and smoking status were measured in 2017 at time of survey. Gravidity was assessed for each pregnancy. | | | | |

| **Supplemental Table 6.** Associations between occupational factors and risk of miscarriage adjusted for age at survey, among 1,074 firefighters and 1,864 pregnancies^a,b^ | | | | |
| --- | --- | --- | --- | --- |
|  | Miscarriages  N | | RR (95% CI)  Model 1^c^ | RR (95% CI)  Model 2^d^ |
| **Firefighter subgroups** |  | | | |
| Employment, stratified by wildland firefighter status |  |  |  |  |
| Structural | No | Yes |  |  |
| Career | 891 | 281 | 1.00 (Ref.) | 1.00 (Ref.) |
| Volunteer | 140 | 64 | 1.41 (1.11 - 1.79) | 1.45 (1.14 - 1.85) |
| Wildland/WUI |  |  |  |  |
| Career | 388 | 41 | 1.00 (Ref.) | 1.00 (Ref.) |
| Volunteer | 38 | 18 | 3.09 (1.76 - 5.42) | 2.66 (1.37 - 5.16) |
| **Work practices** |  | | | |
| Shift schedule of career firefighters, stratified by age at pregnancy |  |  |  |  |
| <35 years old | No | Yes |  |  |
| Less than 24 hr on shift | 232 | 69 | 1.00 (Ref.) | 1.00 (Ref.) |
| 24 or more hr on shift | 772 | 144 | 0.67 (0.50 - 0.90) | 0.73 (0.54 - 0.98) |
| 35+ years old |  |  |  |  |
| Less than 24 hr on shift | 64 | 18 | 1.00 (Ref.) | 1.00 (Ref.) |
| 24 or more hr on shift | 211 | 89 | 1.29 (0.84 - 2.00) | 1.19 (0.76 - 1.88) |
| Worked fire/rescue calls at pregnancy start | No | Yes |  |  |
| No | 138 | 58 | 1.00 (Ref.) | 1.00 (Ref.) |
| Yes | 1250 | 340 | 0.81 (0.60 - 1.09) | 0.80 (0.60 - 1.08) |
| RR= Relative risk; 95% CI= 95% confidence interval; BMI= Body mass index (kg/m^2^); WUI= wildland urban interface  ^a^ Generalized estimating equations models with Poisson distribution and sandwich variance estimators were used to estimate risk ratios and 95% CIs.  ^b^ 76 pregnancies had missing information for wildland status (3), shift schedule (5), and fire/rescue calls (68) and were not included in those models.  ^c^ Model 1 is adjusted for age at pregnancy, and (age at pregnancy)^2^.  ^d^ Model 2 is additionally adjusted for age at survey (years), highest education completed (some college/at least college degree), gravidity (yes/no), BMI (<30 kg/m^2^ / ≥30 kg/m^2^), and smoking status (current or former/never). Education, BMI, and smoking status were measured in 2017 at time of survey. Gravidity was assessed for each pregnancy. | | | | |
